# Estimating Benzathine Penicillin Need for the Treatment of Pregnant Women Diagnosed with Syphilis during Antenatal Care in High-Morbidity Countries

**DOI:** 10.1371/journal.pone.0159483

**Published:** 2016-07-19

**Authors:** Melanie M. Taylor, Stephen Nurse-Findlay, Xiulei Zhang, Lisa Hedman, Mary L. Kamb, Nathalie Broutet, James Kiarie

**Affiliations:** 1 Centers for Disease Control and Prevention, Division of STD Prevention, Atlanta, GA, United States of America; 2 Department of Reproductive Health, World Health Organization, Geneva, Switzerland; 3 Department of Essential Medicines and Health Products, World Health Organization, Geneva, Switzerland; 4 Centre for Tuberculosis Control, Shandong Provincial Chest Hospital, Jinan, China; University of British Columbia, CANADA

## Abstract

**Background:**

Congenital syphilis continues to be a preventable cause of global stillbirth and neonatal morbidity and mortality. Shortages of injectable penicillin, the only recommended treatment for pregnant women and infants with syphilis, have been reported by high-morbidity countries. We sought to estimate current and projected annual needs for benzathine penicillin in antenatal care settings for 30 high morbidity countries that account for approximately 33% of the global burden of congenital syphilis.

**Methods:**

Proportions of antenatal care attendance, syphilis screening coverage in pregnancy, syphilis prevalence among pregnant women, and adverse pregnancy outcomes due to untreated maternal syphilis reported to WHO were applied to 2012 birth estimates for 30 high syphilis burden countries to estimate current and projected benzathine penicillin need for prevention of congenital syphilis.

**Results:**

Using current antenatal care syphilis screening coverage and seroprevalence, we estimated the total number of women requiring treatment with at least one injection of 2.4 MU of benzathine penicillin in these 30 countries to be 351,016. Syphilis screening coverage at or above 95% for all 30 countries would increase the number of women requiring treatment with benzathine penicillin to 712,030. Based on WHO management guidelines, 351,016 doses of weight-based benzathine penicillin would also be needed for the live-born infants of mothers who test positive and are treated for syphilis in pregnancy. Assuming availability of penicillin and provision of treatment for all mothers diagnosed with syphilis, an estimated 95,938 adverse birth outcomes overall would be prevented including 37,822 stillbirths, 15,814 neonatal deaths, and 34,088 other congenital syphilis cases.

**Conclusion:**

Penicillin need for maternal and infant syphilis treatment is high among this group of syphilis burdened countries. Initiatives to ensure a stable and adequate supply of benzathine penicillin for treatment of maternal syphilis are important for congenital syphilis prevention, and will be increasingly critical in the future as more countries move toward elimination targets.

## Introduction

Syphilis infection can be transmitted from mother to infant during pregnancy resulting in congenital syphilis. Injectable penicillin is the only recommended treatment for syphilis occurring in pregnant women to prevent congenital syphilis and for infants born with congenital syphilis [1,2]. Maternal syphilis can result in adverse birth outcomes due to congenital syphilis in over half of untreated pregnancies. These adverse birth outcomes include: prematurity/low-birth weight, congenital deformities (neurological, bone, and organ damage), stillbirth, and neonatal death [3]. Provided early in pregnancy, penicillin can prevent mother-to-child transmission of syphilis and related adverse birth outcomes [4].

WHO has received reports of stock outs and shortages of injectable benzathine penicillin from multiple countries, many with a high burden of maternal and congenital syphilis [5,6]. Benzathine penicillin, used to treat syphilis, is an older, generic, injectable medication that has a limited manufacturing base. Each of these factors may reduce benzathine penicillin availability. [7]. One injection of 2.4 million units of intramuscular benzathine penicillin is recommended for pregnant women with early stage syphilis and three injections spaced by one week apart are recommended for late or unknown stage syphilis [1]. Limited data is available on the use of non-penicillin alternative therapies in pregnancy [2,8]. Azithromycin does not cross the placenta in adequate amounts to treat a syphilis-infected foetus [9], and reports of adverse birth outcomes due to congenital syphilis have been reported among pregnant women treated for syphilis with azithromycin [10]. Syphilis treatment failure due to the emergence of azithromycin resistance has been reported in some regions [2]. Ceftriaxone crosses the placental barrier but the optimal dose and duration of therapy for pregnant women is unknown and displacement of bilirubin from albumin-binding sites may increase the risk of kernicterus in newborns [8, 11]. Tetracyclines are contraindicated during pregnancy [2]. Concerns for untreated or inadequately treated maternal syphilis resulting in adverse pregnancy outcomes call for increased awareness and vigilance to ensure a stable supply of benzathine penicillin.

The World Health Organization (WHO) has established country targets for validation of elimination of mother-to-child transmission of syphilis which include: (1) at least 95% of pregnant women attend antenatal care (ANC), (2) at least 95% of pregnant women receive syphilis screening during ANC and (3) at least 95% of syphilis seropositive pregnant women receive adequate treatment, defined as at least 2.4 mu intramuscular benzathine penicillin G [1, 12]. Increasing syphilis screening during antenatal care to achieve elimination targets will involve increased demand for benzathine penicillin. We sought to estimate the current and projected annual number of benzathine penicillin doses needed for pregnant women who test positive for syphilis and their infants in 30 high-morbidity countries to help manufacturers and countries prepare for full scale antenatal syphilis screening and treatment programs.

## Methods

In projecting global needs for injectable penicillin, we focused on the countries that accounted for a high number of congenital syphilis cases or have been identified by WHO as priority settings for congenital syphilis elimination [13]. We used Global AIDS Response Progress Reporting (GARPR) data [14,15] reported to WHO by countries on syphilis screening coverage and prevalence among pregnant women attending ANC.

### Selection of the 30 high syphilis burden countries

The 12 high-burden countries identified as priority settings for elimination of congenital syphilis were included [13]. These 12 countries represent four WHO regions of Africa (Central African Republic, Ghana, Madagascar, Mozambique, Tanzania, Zambia), the Americas (Honduras, Uruguay), Western Asia and the Pacific (China, Papua New Guinea) and Southeast Asia (Indonesia, Myanmar). We also included data from 14 countries reporting ANC syphilis screening coverage of at least 10% and syphilis seroprevalence of at least 1% for GARPR reporting year 2014 (Argentina, Bolivia, Burkina Faso, Chad, Democratic Republic of Congo (DRC), Haiti, Kenya, Liberia, Mali, Mongolia, Nigeria, South Africa, Swaziland, Uganda). Finally, we included four countries with 2012 birth estimates >2 million [16] but reported syphilis seroprevalence less than 1% (Brazil, Ethiopia, India, Bangladesh) using the most recently reported GARPR data. [14]. This selection process resulted in 30 countries used in the analysis, representing 33% of syphilis-associated adverse pregnancy outcomes globally based upon the most recent (2012) WHO estimates [16].

### Estimates of syphilis diagnosed during pregnancy

Number of births by country was obtained from United Nations, Department of Economic and Social Affairs: *World Fertility Data (2012)* [17]. In order to estimate number of pregnancies by country, number of births by country [17] was adjusted for all-cause stillbirth by adding a stillbirth rate of 18.4 per 1,000 births according to recently published estimates to the number of live births [18]. Estimates of women attending antenatal care (ANC) from selected countries were obtained from the WHO Global Health Observatory [19]. The number of women undergoing syphilis testing during ANC, the syphilis seropositivity estimates, and syphilis test types used were obtained from the 2014 GARPR report. [14].

### Estimates of current and projected need for benzathine penicillin for treatment of pregnant women with syphilis

Using country-reported ANC syphilis seroprevalence, we calculated the number of pregnant women with a positive syphilis test (treponemal, non-treponemal, non-treponemal with treponemal confirmation, or unknown test type) to estimate the number of benzathine penicillin doses needed to prevent mother-to-child transmission of syphilis. The WHO recommended treatment regimen for early infectious syphilis (primary, secondary, early latent) is one intramuscular injection of 2.4 million units of benzathine penicillin [1]. We assumed that each seroreactive woman would receive at least one injectable dose of 2.4 million units of benzathine penicillin early in pregnancy for treatment of early infectious syphilis and to prevent mother-to-child transmission. Estimates of doses needed for treatment of late stage or unknown stage syphilis were not included due to lack on information on syphilis stage at diagnosis.

Projected needs for benzathine, anticipating that these 30 countries achieve 95% syphilis screening coverage of pregnant women during ANC, were calculated by replacing current screening coverage with 95% for each country reporting a screening coverage less than 95%. For countries with a screening coverage rate at or above 95%, current values were used.

### Estimated need for benzathine penicillin to treat live-born infants of women with syphilis

WHO treatment guidelines include the recommendation that *all* infants born to syphilis seropositive mothers receive treatment with a single intramuscular dose of 50,000 IU/kg/ of benzathine penicillin whether or not the mothers were appropriately treated during pregnancy, regardless of the foetal gestational age at time of treatment [1, 20]. Estimates of penicillin need for live-born infants born to mothers with syphilis assumed 100% treatment coverage of syphilis-seropositive antenatal women and 100% infant survival to treatment.

### Estimates of adverse birth outcomes with and without maternal treatment

Estimates of the number of women with probable active syphilis, i.e., seroreactivity in screening and confirmatory tests representing infection that could be transmitted from mother to infant during pregnancy, were calculated based upon on a recent meta-analysis [21]. Specifically, a correction factor of 52.2% was applied for countries using non-treponemal testing only, 53.6% for countries using only treponemal testing, and 68.6% for countries not reporting test type [21]. For countries reporting use of both non-treponemal and treponemal testing, 100% of seroreactive women were considered probable active syphilis.

Risk estimates of adverse birth outcomes resulting from untreated probable active maternal syphilis were based on a meta-analysis [3] as follows: any syphilis-associated adverse pregnancy outcomes (52% of untreated syphilis pregnancies), stillbirth (21%) neonatal death (9%), premature delivery/low birth weight (14.1%), and other clinical congenital syphilis infections in live born infants (16%).

Estimates of number of adverse birth outcomes averted with treatment of pregnant women with syphilis were calculated using data from a review and meta-analysis evaluating the anticipated reduction in adverse birth outcomes with maternal treatment with benzathine penicillin [4]. Although clinical data and previous studies have identified maternal treatment prior to the third trimester is important in preventing syphilis-associated adverse pregnancy outcomes [22, 23], penicillin treatment is recommended for all seroreactive pregnant women regardless of the foetal gestational age at the time maternal infection was detected. We assumed penicillin was needed for all women identified as having a positive syphilis test result during ANC, regardless of the gestational week of testing. We also assumed 100% treatment of these women estimated as having probable active syphilis during pregnancy. In order to calculate estimates of averted adverse pregnancy outcomes following maternal syphilis treatment, we used previously published estimates by Blencowe et al. We calculated global estimates of pregnancy outcome averted for the following: any adverse pregnancy outcome (84% averted), stillbirth (82% averted), neonatal death (80% averted), prematurity/low birth weight (64% averted) and other clinical congenital syphilis (97% averted) [4].

Projections of the number of adverse pregnancy outcomes averted with increases in ANC syphilis screening coverage to 95% and treatment coverage of 100% were included according to previously described methods.

## Results

### Estimates of syphilis diagnosed during pregnancy among women attending ANC and estimated current and projected need for benzathine penicillin

Among the 30 countries in this analysis, attendance at one or more ANC visits ranged from 33.9% (Ethiopia) to 98.7% (Mongolia). The percent of women screened for syphilis during ANC ranged from 1.2% (Indonesia) to 100% (Mongolia). Only 7 (28%) countries reported ANC syphilis screening of ≥95%. Syphilis seroprevalence ranged from 0.1% (Honduras) to 11.3% (Liberia). The countries with the highest syphilis seroprevalence were Liberia (11.3%), Mali (9.5%), and Central African Republic (CAR) (7.6%). The country with the largest number of pregnant women seropositive for syphilis was the Democratic Republic of the Congo (DRC) (n = 66,985), followed by Tanzania (n = 53,624) and China (n = 30,882). The total number of doses of 2.4 MU of benzathine penicillin needed to treat pregnant women with syphilis in these 30 countries was 351,016 (Table 1), with regional estimates calculated at 244,231 doses for the 17 countries in the Africa Region, 38,724 doses for the 6 countries in the Americas region, and 68,061 doses for the 7 countries in the Asia/Pacific region (Table 1).

**Table 1:** Estimated annual needs of benzathine penicillin for pregnant women with syphilis in 30 high morbidity countrie

Country	UN Birth (2012)	Number of stillbirths	Number of pregnancies	Percent pregnant women attending ≥ 1 ANC visit	Number of pregnant women attending ≥ 1 ANC visit	Percent pregnant women tested for syphilis	Number of pregnant women tested for syphilis	Antenatal syphilis prevalence (%)	Number of pregnant women testing positive for syphilis	Estimated benzathine penicillin doses needed to treat syphilis-positive pregnant women	Doses needed with improvement to 95% screening coverage	Additional doses needed with 95% syphilis screening coverage
			A	B	C	D	E	F	G	H	J	K
	UN Birth	Birth x0.0184 (SB)	Birth +Stillbirth	Enter %	C = AxB	Enter %	E = C xD	Enter %	G = E x F	H = G	Cx0.95 x F	K-J
**Africa**												
Burkina Faso	658,561	12,118	670,679	94.9	636,474	100	636,474	1.2	7,638	7,638	7,638	0
Central African Republic*	116,036	2,135	118,171	54.6	64,521	35.8	23,099	7.6	1,755	1,755	4,658	2,903
Chad	408,264	7,512	415,776	42.6	177,121	100	177,121	3.4	6,022	6,022	6,022	0
Democratic Republic of Congo	2,532,245	46,593	2,578,838	89.2	2,300,324	72.8	1,674,636	4	66,985	66,985	87,412	20,427
Ethiopia	2,800,977	51,538	2,852,515	33.9	967,003	28.1	271,728	0.9	2,446	2,446	8,268	5,822
Ghana*	699,542	12,872	712,414	96.4	686,767	33.9	232,814	1.5	3,492	3,492	9,786	6,294
Kenya	1,304,487	24,003	1,328,490	91.5	1,215,568	70.3	854,544	1.4	11,964	11,964	16,167	4,203
Liberia	140,131	2,578	142,709	95.9	136,858	11.1	15,191	11.3	1,717	1,717	14,692	12,975
Madagascar*	652,846	12,012	664,858	82.1	545,849	30.3	165,392	5.9	9,758	9,758	30,595	20,837
Mali	666,216	12,258	678,474	74.2	503,428	26.7	134,415	9.5	12,769	12,769	45,434	32,665
Mozambique*	973,056	17,904	990,960	90.6	897,810	46.3	415,686	4.1	17,043	17,043	34,970	17,927
Nigeria	5,966,230	109,779	6,076,009	60.6	3,682,061	14.9	548,627	1.5	8,229	8,229	52,469	44,240
South Africa	1,059,881	19,502	1,079,383	97.1	1,048,081	74.5	780,820	1.6	12,493	12,493	15,931	3,438
Swaziland	34,363	632	34,995	96.8	33,875	97.8	33,130	3.3	1,093	1,093	1,093	0
Tanzania*	1,610,398	29,631	1,640,029	87.8	1,439,946	98	1,411,147	3.8	53,624	53,624	53,624	0
Uganda	1,407,179	25,892	1,433,071	94.9	1,359,984	17.2	233,917	6.7	15,672	15,672	86,563	70,891
Zambia*	512,305	9,426	521,731	95.7	499,297	50.2	250,647	4.6	11,530	11,530	21,819	10,290
**Regional Total**	**21,542,717**	**396,386**	**21,939,103**		**16,194,966**		**7,859,388**		**244,231**	**244,231**	**497,142**	**252,911**
**Americas**												
Argentina	756,176	13,914	770,090	98.1	755,458	86.5	653,471	1.2	7,842	7,842	8,612	771
Bolivia	237320	4,367	241,687	85.8	207,367	69.1	143,291	1.4	2,006	2,006	2,758	752
Brazil	3,141,309	57,800	3,199,109	96.0	3,071,145	89.5	2,748,675	0.8	21,989	21,989	23,341	1,351
Haiti	261,639	4,814	266,453	84.5	225,153	68.4	154,005	3.9	6,006	6,006	8,342	2,336
Honduras*	180,042	3,313	183,355	94.2	172,720	56.6	97,760	0.1	98	98	164	66
Uruguay*	53,199	979	54,178	97.4	52,769	98.9	52,189	1.5	783	783	783	0
**Regional Total**	**4,629,685**	**85,186**	**4,714,871**		**4,484,612**		**3,849,389**		**38,724**	**38,724**	**44,000**	**5,276**
**Asia/Pacific**												
Bangladesh	3,669,345	67,516	3,736,861	58.7	2,193,537	58.3	1,278,832	0.5	6,394	6,394	10,419	4,025
China*	16,040,000	295,136	16,335,136	95.0	15,518,379	99.5	15,440,787	0.2	30,882	30,882	30,882	0
India	27,063,977	497,977	27,561,954	75.1	20,699,028	65.1	13,475,067	0.2	26,950	26,950	39,328	12,378
Indonesia*	4,805,298	88,417	4,893,715	95.7	4,683,286	1.2	56,199	1.7	955	955	75,635	74,680
Mongolia	63,270	1,164	64,434	98.7	63,597	97.2	61,816	2.6	1,607	1,607	1,607	0
Myanmar*	811,644	14,934	826,578	83.1	686,887	10	68,689	0.7	481	481	4,568	4,087
Papua New Guinea*	197,492	3,634	201,126	66.0	132,743	8.9	11,814	6.7	792	792	8,449	7,658
**Regional Totals**	**52,651,026**	**968,779**	**53,619,805**		**43,977,456**		**30,393,205**		**68,061**	**68,061**	**170,888**	**102,827**
**30-Country Totals**	**78,823,428**	**1,450,351**	**80,273,779**		**64,657,035**		**42,101,982**		**351,016**	**351,016**	**712,030**	**361,014**

*WHO investment case country

### Projections of penicillin need if countries were to achieve 95% syphilis screening coverage

Projections of penicillin need if countries were to achieve 95% syphilis screening coverage at current ANC care attendance levels are presented in Table 1. Improving syphilis screening coverage to at least 95% in these 30 countries would increase overall benzathine penicillin need from 351,016 to 712,030 doses (361,014 dose increase) for pregnant women.

With recommended screening rates, countries that would experience the greatest increase in benzathine penicillin need with expanded syphilis screening to 95% include: Indonesia with 74,680 more benzathine penicillin doses needed (from 955 to 75,635 doses), Uganda with 70,891 more doses needed (from 15,672 to 86,563 doses), Nigeria, 44,240 more doses needed (from 8,229 to 52,469 doses) and Mali, 32,665 more doses needed (from 12,769 to 45,434 doses) (Table 1).

### Estimated need for paediatric-dosed benzathine penicillin to treat live born infants of women with syphilis

Among these 30 countries, at current syphilis screening coverage, 351,016 doses of paediatric-dosed benzathine penicillin treatment would be needed to treat live born infants of women testing positive for syphilis during pregnancy. The countries with the highest annual number of infants requiring penicillin treatment were DRC (66,985 infants), Tanzania (53,624 infants) and China (30,882 infants) (Table 2).

**Table 2:** Estimated paediatric benzathine penicillin regimens needed for treatment of infants exposed to syphilis during pregnancy in 30 high-morbidity countries.

Country	Number of pregnant women testing positive for syphilis	Estimated paediatric benzathine penicillin doses needed assuming 100% maternal treatment and 100% infant survival	Number of pregnant women testing positive for syphilis assuming 95% maternal screening coverage	Estimated paediatric benzathine penicillin doses needed with 95% maternal screening coverage, 100% maternal treatment, and 100% infant survival	Additional paediatric doses needed with 95% maternal syphilis screening coverage
	A	B	D	E	F
	Table 1	A	Table 1	D	E-D
**Africa**					
Burkina Faso	7,638	7,638	7,638	7,638	0
Central African Republic	1,755	1,755	4,658	4,658	2,903
Chad	6,022	6,022	6,022	6,022	0
Democratic Republic of Congo	66,985	66,985	87,412	87,412	20,427
Ethiopia	2,446	2,446	8,268	8,268	5,822
Ghana	3,492	3,492	9,786	9,786	6,294
Kenya	11,964	11,964	16,167	16,167	4,203
Liberia	1,717	1,717	14,692	14,692	12,975
Madagascar	9,758	9,758	30,595	30,595	20,837
Mali	12,769	12,769	45,434	45,434	32,665
Mozambique	17,043	17,043	34,970	34,970	17,927
Nigeria	8,229	8,229	52,469	52,469	44,240
South Africa	12,493	12,493	15,931	15,931	3,438
Swaziland	1,093	1,093	1,093	1,093	0
Tanzania	53,624	53,624	53,624	53,624	0
Uganda	15,672	15,672	86,563	86,563	70,891
Zambia	11,530	11,530	21,819	21,819	10,290
**Regional Totals**	**244,231**	**244,231**	**497,142**	**497,142**	**252,911**
**Americas**					
Argentina	7,842	7,842	8,612	8,612	771
Bolivia	2,006	2,006	2,758	2,758	752
Brazil	21,989	21,989	23,341	23,341	1,351
Haiti	6,006	6,006	8,342	8,342	2,336
Honduras	98	98	164	164	66
Uruguay	783	783	783	783	0
**Regional Totals**	**38,724**	**38,724**	**44,000**	**44,000**	**5,276**
**Asia/Pacific**					
Bangladesh	6,394	6,394	10,419	10,419	4,025
China	30,882	30,882	30,882	30,882	0
India	26,950	26,950	39,328	39,328	12,378
Indonesia	955	955	75,635	75,635	74,680
Mongolia	1,607	1,607	1,607	1,607	0
Myanmar	481	481	4,568	4,568	4,087
Papua New Guinea	792	792	8,449	8,449	7,658
**Regional Totals**	**68,061**	**68,061**	**170,888**	**170,888**	**102,827**
**30-Country Totals**	**351,016**	**351,016**	**712,030**	**712,030**	**361,014**

Projections of benzathine penicillin doses needed for infants if countries were to achieve 95% syphilis screening coverage at current antenatal care attendance levels are presented in Table 2. Improving syphilis screening coverage to at least 95% in these 30 countries would increase overall benzathine penicillin need for infants from 351,016 to 712,030 doses (361,014 dose increase).

### Estimates of adverse birth outcomes and those averted with maternal treatment

Test type correction factors along with probable active syphilis infections for each country are displayed in Table 3. The total number of probable active syphilis infections among pregnant women in these 30 countries was 219,638. Assuming no maternal treatment was available, overall estimated adverse birth outcomes due to congenital syphilis among these probable active syphilis cases were 114,212 overall, (46,124 stillbirths, 19,767 neonatal deaths, 13,178 premature delivery/low birth weight, and 35,142 clinical congenital syphilis infections)(Table 3).

**Table 3:** Estimates of syphilis-associated adverse birth outcomes and estimates of those averted through maternal treatment with benzathine penicillin in 30 high-morbidity countries.

	Current ANC Syphilis Screening Coverage													95% Screening Coverage	
Region/Country	Number of women testing positive for syphilis	Syphilis diagnostic test type correction factor (%)	Number of pregnant women with probable active syphilis	Any adverse birth outcomes expected without treatment (52%)	Adverse birth outcomes averted with treatment (84%)	Stillbirths expected without treatment (21%)	Stillbirths averted with treatment (82%)	Neonatal deaths expected without treatment (9%)	Neonatal deaths averted with treatment (80%)	Premature/low birth weight births expected without treatment (6%)	Premature/ low birth weight births averted with treatment (64%)	Clinical infections in live born infants expected without treatment (16%)	Clinical infections in live born infants averted with treatment (97%)	Number of pregnant women with probable active syphilis	Number of adverse pregnancy outcomes averted all women treated (84%)
	A	B	C	D	E	F	G	H	I	J	K	L	M	N	O
	Table 1		AxB/100	C x 0.52	D x 0.84	C x 0.21	F x 0.82	C x 0.09	H x 0.80	C x 0.06	J x 0.64	C x 0.16	L x 0.97	95% Screen	N = M x0.84
**Africa**															
Burkina Faso	7,638	52.2	3,987	2,073	1,741	837	687	359	287	239	153	638	619	3,987	3,349
Central African Republic	1,755	53.6	941	489	411	198	162	85	68	56	36	151	146	2,497	2,097
Chad	6,022	68.6	4,131	2,148	1,804	868	711	372	297	248	159	661	641	4,131	3,470
Democratic Republic of Congo	66,985	52.2	34,966	18,183	15,273	7,343	6,021	3,147	2,518	2,098	1,343	5,595	5,427	45,629	38,329
Ethiopia	2,446	52.2	1,277	664	558	268	220	115	92	77	49	204	198	4,316	3,625
Ghana	3,492	53.6	1,872	973	818	393	322	168	135	112	72	299	291	5,246	4,406
Kenya	11,964	52.2	6,245	3,247	2,728	1,311	1,075	562	450	375	240	999	969	8,439	7,089
Liberia	1,717	68.6	1,178	612	514	247	203	106	85	71	45	188	183	10,079	8,466
Madagascar	9,758	68.6	6,694	3,481	2,924	1,406	1,153	602	482	402	257	1,071	1,039	20,988	17,630
Mali	12,769	68.6	8,760	4,555	3,826	1,840	1,508	788	631	526	336	1,402	1,360	31,168	26,181
Mozambique	17,043	68.6	11,692	6,080	5,107	2,455	2,013	1,052	842	701	449	1,871	1,815	23,989	20,151
Nigeria	8,229	68.6	5,645	2,936	2,466	1,186	972	508	406	339	217	903	876	36,204	30,411
South Africa	12,493	52.2	6,521	3,391	2,849	1,369	1,123	587	470	391	250	1,043	1,012	8,316	6,985
Swaziland	1,093	68.6	750	390	328	158	129	68	54	45	29	120	116	750	630
Tanzania	53,624	68.6	36,786	19,129	16,068	7,725	6,335	3,311	2,649	2,207	1,413	5,886	5,709	36,786	30,900
Uganda	15,672	53.6	8,400	4,368	3,669	1,764	1,447	756	605	504	323	1,344	1,304	46,398	38,974
Zambia	11,530	53.6	6,180	3,214	2,699	1,298	1,064	556	445	371	237	989	959	11,695	9,824
**Regional Totals**	**244,231**		**146,025**	**75,933**	**63,784**	**30,665**	**25,145**	**13,142**	**10,514**	**8,761**	**5,607**	**23,364**	**22,663**	**300,617**	**252,518**
**Americas**															
Argentina	7,842	52.2	4,093	2,129	1,788	860	705	368	295	246	157	655	635	4,496	3,776
Bolivia	2,006	100	2,006	1,043	876	421	345	181	144	120	77	321	311	2,758	2,317
Brazil	21,989	52.2	11,478	5,969	5,014	2,410	1,977	1,033	826	689	441	1,837	1,781	12,184	10,234
Haiti	6,006	68.6	4,120	2,143	1,800	865	710	371	297	247	158	659	639	5,723	4,807
Honduras	98	68.6	67	35	29	14	12	6	5	4	3	11	10	113	95
Uruguay	783	68.6	537	279	235	113	92	48	39	32	21	86	83	537	451
**Regional Totals**	**38,724**		**22,302**	**11,597**	**9,742**	**4,683**	**3,840**	**2,007**	**1,606**	**1,338**	**856**	**3,568**	**3,461**	**25,810**	**21,680**
**Asia/Pacific**															
Bangladesh	6,394	53.6	3,427	1,782	1,497	720	590	308	247	206	132	548	532	5,585	4,691
China	30,882	100	30,882	16,058	13,489	6,485	5,318	2,779	2,223	1,853	1,186	4,941	4,793	30,882	25,941
India	26,950	52.2	14,068	7,315	6,145	2,954	2,423	1,266	1,013	844	540	2,251	2,183	20,529	17,245
Indonesia	955	100	955	497	417	201	165	86	69	57	37	153	148	75,635	63,533
Mongolia	1,607	68.6	1,103	573	482	232	190	99	79	66	42	176	171	1,103	926
Myanmar	481	68.6	330	172	144	69	57	30	24	20	13	53	51	3,134	2,632
Papua New Guinea	792	53.6	546	284	239	115	94	49	39	33	21	87	85	5,796	4,869
**Regional Totals**	**68,061**		**51,311**	**26,682**	**22,413**	**10,775**	**8,836**	**4,618**	**3,694**	**3,079**	**1,970**	**8,210**	**7,963**	**142,663**	**119,837**
**30-Country Totals**	**351,016**		**219,638**	**114,212**	**95,938**	**46,124**	**37,822**	**19,767**	**15,814**	**13,178**	**8,434**	**35,142**	**34,088**	**469,089**	**394,035**

Assuming availability of penicillin and treatment for all mothers diagnosed with syphilis and using current syphilis screening coverage, an estimated 95,938 adverse birth outcomes overall would be prevented. This would include 37,822 stillbirths, 15,814 neonatal deaths, 8,434 prematurity/low birth weight, and 34,088 congenital syphilis cases (Table 3).

Projections of overall adverse birth outcomes averted if these countries were to achieve 95% syphilis screening and 100% treatment coverage of women with probable active syphilis are presented in Table 3. At current ANC attendance levels and ANC seroprevalence, 394,035 adverse pregnancy outcomes could be averted with screening improvements to 95% of ANC attendees and 100% treatment coverage (Table 3).

## Discussion

In response to reports of benzathine penicillin shortage, we analysed ANC syphilis surveillance data from 30 high-morbidity countries representing only one-third of estimated syphilis-associated adverse birth outcomes to inform efforts to ensure stable supplies of benzathine penicillin. Using current ANC attendance and syphilis screening coverage, we estimated an immediate need for approximately 350,000 doses of benzathine penicillin to treat pregnant women seropositive for syphilis and an additional 350,000 doses of paediatric-dosed benzathine penicillin to treat infants exposed to syphilis during pregnancy. Current need is greatest in Africa, followed by Asia and the Americas regions. Assuming each of the pregnant women in this analysis were treated with at least 2.4 million units benzathine penicillin as recommended by WHO, more than 95,000 adverse pregnancy outcomes due to maternal syphilis would be averted in these countries.

The WHO initiative to eliminate congenital syphilis calls for at least 95% of pregnant women to receive ANC, at least 95% of these pregnant women to be tested for syphilis during prenatal care and at least 95% of syphilis-infected (seroreactive) pregnant women to receive treatment [12]. Although ANC attendance was 95% or greater for approximately half of these 30 countries, less than one-third of countries performed syphilis screening for ≥95% of ANC populations. As countries work to achieve the elimination targets, benzathine penicillin need will increase. Our projections suggest an approximate doubling of annual penicillin need for pregnant women and infants if these 30 countries were to strive for and achieve at least 95% coverage for syphilis screening, a value that would be higher with improvements in ANC coverage and greatest in Africa.

WHO recommends that all infants born to seropositive mothers receive treatment with a single intramuscular dose of benzathine penicillin regardless of whether the mother received treatment during pregnancy [1]. These paediatric penicillin estimates demonstrate need for penicillin formulations, including benzathine that can be reliably weight-adjusted to assure proper dosing. Current pre-filled syringes offer the dosing options of 600 thousand or 1.2 or 2.4 million units of benzathine penicillin, amounts not easily weight-adjustable for newborn infant treatment [24–25]. Efforts to engage manufacturers regarding the supply and dosing needs of these high-morbidity countries are needed to promote production of benzathine penicillin that can be correctly weight-dosed for infants at affordable cost.

A single dose of benzathine penicillin treatment ends infectivity in adults, and if received sufficiently early in pregnancy will treat the mother and prevent or treat congenital syphilis in the foetus. Penicillin treatment is safe and allergy is a very rare event (4 cases/million). [26] Our adverse birth outcome estimates among women with syphilis necessarily assumes that these women either received no treatment or were treated too late in pregnancy to prevent mother-to-child transmission of syphilis. Published studies suggest that early detection and treatment of syphilis in pregnancy is critical as delayed treatment, after the first trimester, can still result in syphilis-associated adverse outcomes [22, 23]. This was corroborated by a recent analysis indicating that among syphilitic women treated in the third trimester, 64.4% of pregnancies had poor pregnancy outcomes compared with 13.3% of pregnancies in syphilitic women treated in the first trimester, and 13.7% of pregnancies in women without syphilis. [27]. In this analysis, projected adverse pregnancy outcomes averted with maternal penicillin treatment were high, particularly among African countries. These estimates of country-level maternal syphilis burden and preventable adverse pregnancy outcomes with treatment of syphilis-infected pregnant women can be used in planning and projecting need for dependable national supplies of benzathine penicillin.

There are limitations in deriving penicillin need from these estimates. Low testing coverage of pregnant women in some countries with high ANC syphilis prevalence underestimates benzathine penicillin need were testing coverage to improve. Also, infants with symptomatic congenital syphilis should receive additional treatment with aqueous crystalline penicillin according to WHO treatment guidelines [1]. We did not generate estimates of need for this formulation of penicillin (aqueous) for infants. We estimated paediatric benzathine penicillin need on the assumptions of 100% treatment of mothers with positive syphilis test results and 100% survival of infants; syphilis-associated stillbirth and neonatal death resulting from no or late maternal treatment would reduce the number of paediatric doses needed [25]. We did not account for treatment needs of pregnant women with late latent stage syphilis which requires more than one dose of benzathine penicillin. We did not address timing of treatment during pregnancy, thus, the number of CS cases averted could be fewer in situations where maternal treatment occurred late in pregnancy, even if the infants could be successfully treated after birth. Penicillin needs estimates for pregnant women and infants were based on maternal syphilis seroprevalence which does not accurately reflect active syphilis infection due to variations in syphilis test type by country. We did not include a sensitivity analysis or any measure of variability. Studies have identified other barriers to treatment not related to penicillin supply [22]. We did not address these. We did not address drug shipment, storage, or supply challenges such as product loss due to expiration or other inventory management issues. Recent syphilis incidence estimates for adults ages 15–49 indicate 5.6 million new syphilis cases occur globally each year [28]. This analysis focused only on syphilis in pregnancy and does not evaluate countries’ needs for benzathine penicillin for treatment of sex partners of syphilis-infected women to prevent reinfection, non-pregnant adults with sexually-acquired syphilis, or for other conditions such as rheumatic heart disease or other streptococcal infections [29].

Of all syphilis-infected persons, pregnant women and their infants are the most vulnerable. Recently reported data indicate that progress has been made since the 2007 launch of the elimination of congenital syphilis initiative; however, the global maternal syphilis burden remains high with more than 900,000 estimated maternal syphilis infections resulting in 350,000 adverse birth outcomes in 2012 [15]. The maternal morbidity due to syphilis in these 30 countries accounts for one-fifth of the estimated global maternal burden and approximately one-third of estimated adverse pregnancy outcomes due to congenital syphilis. Needs estimates of benzathine penicillin can help ensure needed treatment is available for pregnant women and their infants and anticipate increased demand. With development, validation, and expanded use of rapid syphilis tests, including dual rapid HIV/syphilis tests [30], syphilis screening during ANC is expected to increase as is same-day penicillin treatment of pregnant women testing positive for syphilis.

Ensuring a continuous global supply of benzathine penicillin to match increasing global demand is a critical component of preventing infant morbidity and mortality associated with syphilis in pregnancy. These needs estimates provide scope and scale in addressing high-morbidity country needs for benzathine penicillin. Assessing benzathine penicillin need is but one component of a broader evaluation of congenital syphilis prevention through maternal syphilis screening during ANC. WHO, in collaboration with international partners, has spearheaded an initiative to assess global supply, current and projected demand, and production capacity for benzathine penicillin. Further evaluation is needed to identify and address other barriers to treatment of pregnant women with syphilis at local, country, and regional levels.
